# The Relations Existing between Facial Neuralgia, Frontal Headache and Dental Caries

**Published:** 1885-12

**Authors:** H. Boyle Runnalls

**Affiliations:** Saltash


### THE RELATIONS EXISTING BETWEEN FACIAL NEURALGIA, FRONTAL HEADACHE AND DENTAL CARIES.

BY

H. BOYLE RUNNALLS, M.R.C.S.,

*Saltash.*

DURING a comparatively short medical career, I have been astonished at the large number of cases which have come under my care owing their origin either to undeveloped teeth or to teeth undergoing a degenerative process, and that, on referring to my case-book, by finding that the percentage of such cases is on the increase. Some ten years ago I made a collection of human bones and teeth, which I obtained by excavating in the sand-hills on the west side of Crantock, a small village on the north coast of Cornwall. There is no record existing that will account for the presence of such a large quantity of bones there of both old and young human beings. The skeletons are found lying in all directions, and not separated from each other by anything but sand, and I could not find any relic to assist me in ascertaining when and why they were placed there. I obtained several hundred of the teeth, most of which were scattered loose; but some I found *in situ*, which fell out as soon as the surrounding sand was removed. All the lower maxillary bones which I was fortunate enough to find intact differed greatly from those now seen, inasmuch as they are very much larger and the fore part squarer. Those that I saw *in situ* contained the teeth in perfect position and condition, with no jambing of tooth upon tooth.

The upper edges of the incisors were regular, and presented no notches or dents. The other bones were correspondingly large, particularly the scapulæ, humeri, and femora. I could not collect sufficient of the latter in a perfect state to take an average length. None of the teeth that I found presented even the slightest appearance of deterioration, although I searched most assiduously to find even one that did. Although noticing the absence of decay in the teeth, and the larger size of the jaw bones, I only attributed it then to a freak of nature; but since I have become better acquainted with the human family, and noticed the great rarity of good teeth and the early appearance of decay, I have begun to think there must be cause and effect to account for the vast difference. Is not this one of the proofs of the gradual change which is taking place in the construction of the human frame? Even in the present day we find those members of the human family who use their teeth for chewing coarse uncooked foods possess masticatory muscles which are prominent and largely developed, and the bones to which they are attached developed in a like manner, and teeth which rarely present any appearance of decay. In the civilised races, who feed upon soft cooked foods, we find that the jaws become smaller and more contracted, with a consequent pressing of tooth upon tooth, and decay appearing at a very early age. The decay increases in the younger members as we ascend in the social scale; but it is difficult to arrive at the actual percentage, for the dentist places an obstacle at so doing by removing some of the teeth of the children of the wealthier classes, which is recognised as a necessity if it is wished to preserve the remainder.

This alteration in the size of the mouth and condition of the teeth, gradual as it is, is accountable for many abnormal gastric conditions, but more particularly for those pains in the regions of the face and cranium, which, I think, are wrongly called "neuralgia," when that term is used to designate a distinct disease, and to be treated as such. Personally, I have never seen a case of "neuralgia" in which I did not find that it was produced by either a carious fang, periostitis of tooth cavity, carious tooth, dead tooth, undeveloped wisdom-tooth, exostosis of fang producing pressure on nerve, hardening of dentine, eventually producing closure of pulp cavity and pressure on the nerve, or too close approximation of the teeth, and which disease always disappeared when these causes were removed. (Of course I do not mean those cases which may arise from pressure of some growth upon a nerve, or where a nerve may be implicated with carious bone or a scar.) I daresay the following clinical records will bring to the readers' recollection many like cases; and my only excuse for so detailing them is to strongly impress the necessity for operative treatment, and not the constant administration of "bromide" and its allies, which often aggravate the dyspeptic symptoms from which the patient suffers:

B. H., female, age 30. When I first saw her she complained of pain in region of the ovaries, and constant pain in forehead; said she was very subject to "hysteria." As she told me she had an offensive vaginal discharge, I examined the canal, but found nothing but what ordinary cleanliness would remove. On examining her mouth I found the teeth were artificial, and had been fitted on the stumps of carious teeth. I advised removal of all the stumps, which she allowed me to do, and from that time all the aches and pains disappeared, and the hysteria also.

She had had a varied experience of medical skill for seven years, and must have taken in that time gallons of physic.

B. S., female, age 28. Complained of earache when I first saw her; otherwise appeared to be perfectly healthy. Could not discover any cause in the external meatus to account for the pain. The throat was in a normal condition, and the teeth fairly good. Not feeling satisfied as to the cause of the pain, I gave her a mixture of olive oil and opium to drop into the ear, but which produced no benefit. I continued this treatment for some time, when one day she complained of pain over the angle of the jaw on the same side that the ear ached. I carefully examined the teeth and face, and again was baffled as to the cause of it; but a day or so after I found that the tissues behind the last visible molar were swollen. I incised them, which resulted in the discharge of a small quantity of pus. I naturally thought there must be diseased bone somewhere near, and the probe proved that I was right, but not the kind of bone I thought it was. On making freer incisions I found that my carious bone was a carious wisdom-tooth, which had not appeared above the gums. I extracted it, and the earache almost instantly disappeared.

G. T., aged 32, female. When she came for my advice, complained of constant faceache, complicated at times with hysteria. Said she had suffered from it since the age of 19. Had resorted to numberless remedies, but never had any relief. The teeth, to my astonishment, were remarkably good, not one of them presenting any signs of decay. As the wisdom-teeth were not visible, and as she told me that they had never appeared, I made an incision over where they should be, and found the crowns about $\frac{1}{16}$th of an inch below the surface of the gums. Thinking the removal of the tension might give relief, I waited for a fortnight, at the end of which time I extracted both, as the pains in face had not decreased. After their removal all the pains disappeared, and she has never had a return of them.

Hamilton Moore, Esq., practising as a dentist in Paris, informed me that a lady was sent to him by a renowned European oculist, for the purpose of having the stump of the left canine extracted. On its removal the lady immediately exclaimed, "Why, I can see again!" This lady had lost the sight in the left eye for several years, and had begun to lose all hope of its recovery.

I could relate numbers of like cases, and cases of dyspepsia, where ailments all disappeared when the teeth were attended to. It is an invariable rule with me now to carefully examine the teeth when a neuralgic or dyspeptic patient puts in an appearance, and I absolutely refuse to give physic when I find a tooth presents any appearance of caries or retarded growth. I frequently get cases of facial neuralgia where the teeth have been filled, and I always strongly advise their removal, with an invariable result of instant relief, as I have always found that the teeth have been ineffectually filled, either because the whole of the carious tissue had not been removed previous to the tooth being filled, or because there was periostitis of the fang existing with the carious cavity. I remember a case well exemplifying the foregoing. A lady came for my advice as to whether a carious tooth should be removed or filled. By the side of it was another which had been previously filled. From the appearance of the tooth that had been filled, and the condition of the gums around it, I felt convinced that it was the offender. I removed it, and found my surmise was right, there being an extensive accumulation of pus at the bottom of the cavity, and it had welled up into the pulp cavity underneath the filling. The other tooth was filled, and the neuralgia made a speedy exit. No one would dream of giving physic for pain in the knee resulting from hip-joint disease, or pain at top of penis and in the groins resulting from stone in the bladder, and I am of an opinion that it is quite as hopeless to give physic with the intention of curing "facial neuralgia;" but notwithstanding, there are gallons upon gallons of nauseous drugs annually prescribed which may be alphabetically arranged from assafœtida to valerian, when the performance of a quick but painful operation would result in an almost instantaneous cure. Of course I would not extract a tooth, or advise its removal, if I thought the tooth might be saved by being properly filled; but unless the tooth is carefully and tenderly dealt with during the cleaning and filling process, it would be much preferable to have it extracted. There are many dentists, but few good ones—at any rate, that is my experience,—and rather than allow a tooth to be treated as a lump of ivory, forgetting altogether its delicate construction, I should always advise extraction, unless the person's means would allow them to place themselves under the care of a thoroughly scientific man.

